# The Census Report

**Published:** 1921-09-10

**Authors:** 


### THE PUBLIC WELL-BEING.

### The Census Report.

CONSIDERABLE anxiety has been felt as to the broad results of a Census taken so shortly after the most devastating war known in our history. Speaking generally, the result is less unfavourable than has been feared. The total population for Great Britain is 42,767,530. The increase in the past decade is the smallest yet registered. But an increase there is at the rate of 4.7, as compared with 10.4 in 1901-11, and it is perhaps as much as the country could bear. So far as war is concerned the loss of those slain in the service of their country makes but a small impression on the statistics. The loss of their possible offspring is a far more serious factor in the population returns. But the full effect of war upon the country will not be felt for many years to come, when the potential children of the healthiest and hardiest stock in the kingdom should, in normal course, have become fathers in their turn. Yet the recent more reassuring reports of the Registrar-General give rise to the hope that there may be counteracting forces in the race which may obviate the danger ahead. The decline in the number of Londoners may fairly be attributed in part, at any rate, to lack of housing accommodation. The county and City of London contain 38,436 fewer people than in 1911, but office accommodation has been strained to the utmost, and people have been driven further afield for their homes. Greater London shows an increase, though a slight one. Such an increase can only properly follow in the wake of building operations. Where large increases have taken place we suspect a large proportion of over-crowded tenements, and await with interest the further particulars, provided for in the returns, which shall throw light upon numbers in proportion to rooms. Read in comparison with vital statistics in each locality these returns are likely to prove of great value.

The existence of an excess of two million women over males in Great Britain revealed in the returns was long ago forecasted. To every 1,000 males there are 1,101 females in England, 1,009 in Wales, and 1,078 in Scotland. It is well to remember that this preponderance of females is probably purely temporary. Every reduction in the infantile death-rate tends to equalise the proportion of the sexes, for it is in the first year of life that the losses to the male sex are greatest, and more males are born than females. Nevertheless, for this generation the excess of females is likely to continue, and must be met in the public interest by recognition of single women's claim to be self-supporting, and to be treated industrially on an equal basis with men as wage-earners.

Much disquiet is occasioned by the depopulation of the countryside in favour of the towns. The rural districts now contain little more than a fifth of the whole population. In some counties the population has actually declined, and some 79 per cent. of the people of England and Wales are town-dwellers and town workers. Grave as this fact must appear it is by no means certain that it is the result of any more recondite cause than scarcity of accommodation for those who would fain remain on the land or go back to it. It would be difficult to point to any country village containing unoccupied cottages. The causes which promote building syndicates in towns are lacking in country districts. The erection of cottages has long proved a highly unremunerative business even for landowners urgently requiring accommodation for their workpeople, and every year old cottages are falling into an uninhabitable condition. Nothing can stop the gradual inflowing stream of English people to the towns but a recognition of the fact that they cannot remain in the country or return to it unless more cottages, many more, are built in the rural districts.

